# A Highly Effective Protocol for the Rapid and Consistent Induction of Digital Dermatitis in Holstein Calves

**DOI:** 10.1371/journal.pone.0154481

**Published:** 2016-04-27

**Authors:** Adam C. Krull, Vickie L. Cooper, John W. Coatney, Jan K. Shearer, Patrick J. Gorden, Paul J. Plummer

**Affiliations:** 1 Department of Veterinary Diagnostic and Production Animal Medicine, College of Veterinary Medicine, Iowa State University, Ames, Iowa, United States of America; 2 Department of Veterinary Microbiology and Preventative Medicine, College of Veterinary Medicine, Iowa State University, Ames, Iowa, United States of America; University of Minnesota, UNITED STATES

## Abstract

Bovine Digital Dermatitis (DD) is a leading cause of lameness in dairy cattle. DD is reportedly increasing in prevalence in beef cattle feedlots of the US. The exact etiologic agent(s) responsible for the disease have yet to be determined. Multiple studies have demonstrated the presence of a variety of *Treponema* spp. within lesions. Attempts to reproduce clinically relevant disease using pure cultures of these organisms has failed to result in lesions that mirror the morphology and severity of naturally occurring lesions. This manuscript details the systematic development of an experimental protocol that reliably induces digital dermatitis lesions on a large enough scale to allow experimental evaluation of treatment and prevention measures. In total, 21 protocols from five experiments were evaluated on their effectiveness in inducing DD lesions in 126 Holstein calves (504 feet). The protocols varied in the type and concentration of inoculum, frequency of inoculation, duration the feet were wrapped, and type of experimental controls need to validate a successful induction. Knowledge gained in the first four experiments resulted in a final protocol capable of inducing DD lesions in 42 of 44 (95%) feet over a 28 day period. All induced lesions were macroscopically and microscopically identified as clinical DD lesions by individuals blinded to protocols. Lesions were also located at the site of inoculation in the palmer aspect of the interdigital space, and induced clinically measurable lameness in a significant portion of the calves. Collectively these results validate the model and provide a rapid and reliable means of inducing DD in large groups of calves.

## Introduction

Bovine Digital Dermatitis (DD) is a leading cause of lameness in dairy cattle in the United States [1] and is beginning to have an increased prevalence in beef cattle feedlots [2]. DD accounted for 61.8% of the lameness in bred heifers and 49.1% of the lameness in cows in the most recent National Animal Health Monitoring System survey of US dairy farms [1]. Despite over 40 years of research, the identification and cultivation of etiological agent(s) with the ability to consistently recreate clinical disease have largely failed. The initial description of DD as an ulcerative disease of the bovine coronary band occurred at the 8th International Meeting on Diseases of Cattle in Milan, Italy [3]. Some of the first reports describing potential etiologic agents associated with DD were published in 1992, followed by a report describing the isolation and identification of an anaerobic spirochete, believed to be a *Treponema* spp. [4, 5]. A number of additional papers have been published demonstrating the association of the lesions with additional bacteria including *Bacteroides* spp. [6–10] and *Porphyromonas* spp. [9–11], *Campylobacter* spp. [12–16], and *Dichelobacter nodosus* [17–22]. A positive clinical response to topical antimicrobial therapy [23–27] and the lack of viral or fungal DNA from shotgun metagenomics [28] suggests the disease process is bacterial in nature. More recent literature using culture-independent technology suggests the disease process is likely poly-bacterial in nature with multiple *Treponema* spp. involved at various stages of lesion development [6, 28, 29]. This hypothesis is supported by the fact that while several *Treponema* phylotypes are consistently identified in DD lesions [6, 30–34], attempts to induce disease using pure cultures of cultivable *Treponema* spp. have failed to induce significant DD lesions [35]. Additionally, killed vaccines using cultivable spirochetes provide limited protection against DD development [36]. The association of DD lesions with a variety of bacterial agents, the response of the lesions to antibiotics, and the failure to induce or protect from the disease using monovalent vaccines strongly suggests that DD is a polymicrobial disease process [34, 37–39].

Microscopic changes associated with the development of DD have been previously described [14, 17, 40–43]. DD lesions are histopathologically characterized by acute, suppurative inflammation of the epidermis with superficial necrosis and hyperkeratosis [42], along with perivascular aggregations of lymphocytes and plasma cells [7]. A consistent microscopic observation of spirochetes within lesions has been demonstrated by multiple researchers through the use of Hematoxylin and Eosin staining, Warthin–Starry silver staining [44], immunohistochemistry [33, 41], electron microscopy [33, 45], and fluorescence *in situ* hybridization (FISH) [17, 18, 20, 45–49]. Recently a large set of naturally occurring lesions (193 lesions) of various stages was evaluated for pathologic changes associated with developing DD lesions [28] and a histopathologic lesion grading system was developed with three grades of severity that describe the chronicity of disease.

Despite the fact that DD is likely a poly-bacterial disease process, attempts to induce disease with a mixture of cultivable bacterial organisms isolated from natural DD lesions has yet to be attempted. There are two published reports of attempting to induce DD lesions by inoculation of pure growth bacterial cultures. Gomez et al [35] attempted to induce DD with *Treponema* sp. in four yearling Holstein heifers. Only four feet of the four heifers in this induction trial were utilized for attempting to induce with pure cultures of *Treponema* sp. Only one site was considered a successful induction with the lesion described histologically as being similar to a DD lesion, but with a “sparse bacterial mat, light invasion of spirochetes, minimal inflammation, and no ulceration”. The only other attempt at inducing DD lesions with cultivable organisms was in a murine abscess model where multiple isolates of *Treponema* sp. isolated from DD lesions were able to induce abscess formation [50].

Given the unclear etiology of DD, several attempts utilizing DD lesion homogenate as inoculum in an induction model have been undertaken. Gomez et al. [35] tested an experimental model utilizing DD homogenate on six feet from four Holstein yearlings utilizing a complex multi-layered foot wrap and a plastic boot. Four of the six feet were considered to have a lesion consistent with DD in the 63 day protocol. However, the induced lesions were located adjacent to the dew claws and all attempts to induce lesions in the typical DD location [51] near the interdigital fold failed. Read and Walker described the successful induction of 6 calf feet using DD homogenate and a wrap in an abstract at the 47^th^ annual meeting of the American College of Veterinary Pathologists [52]. A full description of the model with morphologic and histologic descriptions of the induced lesions was never published.

Based on the paucity of robust experimental models for DD induction present in the literature, there is a substantial need for the development of an consistent and predictable, experimental model that induces clinically relevant digital dermatitis lesions. Models are important tools for studying and confirming disease etiology, exploring the bacterial and host response to infection, and for conducting controlled infections to evaluate novel therapeutics or vaccine candidates. To the extent that this model could be used to study the protective effects of novel digital dermatitis vaccines, it needs to conform to the guidelines of the USDA APHIS Biologics Regulations and Guidance [53]. The guidelines have several important implications for model design. First, the guidelines require that subjects should be immunologically naïve to the disease prior to enrollment of the study. Given the significant prevalence of DD reported in Holsteins as early as breeding age heifers [54–56], and the lack of validated screening tools that can be used to exclude prior exposure, proving immunologic naïveté can be a challenge in mature cattle. One potential solution is to use young calves with a verifiable disease history. The USDA guidelines also require that efficacy studies include a placebo group in order to calculate the prevented fraction. This requires that a successful protocol have a high rate of success inducing lesions in treatment groups while not inducing disease in the negative control group. Finally, in order to reach statistical significance in an efficacy study, the disease model needs to be easily scaled up to include a large number of animals.

The objective of this project was to develop and validate an induction model that would produce DD lesions in immunologically naïve calves. We hypothesized that inoculation of macerated DD lesion material, collected and handled in a manner to minimize oxidative stress, into a favorable environment of immunologically naive calves would result in consistent induction of clinical disease. Our approach relied on sequential testing of various combinations of inocula, wrap duration and skin abrasion. As improvements in the methodology were made, we also evaluated the use of cocktails of cultivable DD associated organisms for their ability to induce disease. Through systematic evaluation of 21 different protocols we were able to develop a finalized consensus protocol that resulted in a 95% induction rate over a 28-day study.

## Materials and Methods

Holstein dairy calves utilized for this study were approximately 200 pounds, 3 months of age, and weaned a minimum of 30 days. For inclusion in the study, calves needed to be vaccinated for respiratory pathogens prior to arrival, BVD-PI negative and free of antibiotic residue at the start date of the trial. Calves were determined to be free of antibiotic residue as long as any systemic antibiotics given prior to arrival had extended past each drugs slaughter withhold period. Throughout the course of the study, calves were fed a diet that consisted of free-choice hay and a whole corn / protein mix that did not contain antibiotics or ionophores. All calves were deemed to be in good health prior to onset of induction and any animals that required antibiotic treatment were removed from the study analysis. Calves experiencing lameness greater than locomotion score 4 on a standardized 5 point locomotion scoring system [57] on these studies, were evaluated and treated by a veterinarian. All animal procedures and protocols were approved by the Institutional Animal Care and Use Committee of Iowa State University. Calves exhibiting lameness were classified into three categories based on the physical exam performed by the veterinarian: 1) DD lesion associated lameness, 2) wrap associated lameness (i.e. wrap cutting into skin or inducing pain) and 3) lameness unrelated to DD.

To assess pain and/or lameness associated with developing DD lesions on each individual foot, we developed an objective measure for each individual foot. Foot sensitivity was classified as one of five scores: 0) No signs of sensitivity, 1) Holds foot in air when standing still, 2) Favors when walking, 3) Reluctant to bear weight 4) Non-weight bearing. Scoring of foot sensitivity was done daily for the first three weeks of the trial and three times weekly thereafter. Any foot that was given a score of 3 was examined on the tilt table for signs of wrap associated lameness. If the lameness was due to a wrap issue, the wrap was removed and appropriate treatment was initiated. As lameness was one of our measurable outcomes for the induction of DD lesions, if lameness was not associated with a wrap, the wrap was left in place and anti-pain medication was administered (meloxicam 1mg/kg EOD). As long as the lameness was responsive to pain medication and the lameness did not reach the level of non-weight bearing, the DD lesions were allowed to remain untreated until the conclusion of the study. At the conclusion of the study, each foot was determined if they experienced DD associated lameness while on study. Any animal that experience wrap associated lameness and those that received systemic antibiotics were excluded from analysis. We defined “DD associated lameness” as a foot that had a minimum of two observations of sensitivity in which at least one of them was a score of 2 or more.

At the conclusion of the study, calves were treated with between one and six treatments of topical tetracycline until all evidence of visible lesions were healed. Four preliminary studies were conducted to optimize the induction conditions and exact methodologies that led to a final protocol. Table 1 is a summary of the protocols used in each of the experiments with the number of feet, wrap length, pen designations, and type of inocula detailed for each protocol. A detailed materials and methods for the four preliminary experiments are presented as a supplementary file (S1 File) to this manuscript. As experiment 5 represents the final consensus protocol, the methodologies employed in this protocol will be described in detail below.

**Table 1:** A summary of each protocol evaluated during the five experiments.

Experiment	Wrap Length	Protocol	Inoculum	Pen	Protocol Description	# Feet Enrolled
Experiment 1	7 days	1	1	A	No Abrasion with Macerated Lesion Material	20
	7 days	2	1	A	Foot Abrasion with Macerated Lesion Material	20
Experiment 2*	25 days	1	1	B	Controls—Segregated	12
	25 days	2	1	A	Controls—Within Pen	15
	25 days	3	2	A	Macerated Lesion Material + *Treponema phagedenis*	15
	25 days	4	3	A	Macerated Lesion Material + *Dichelobacter nodosus*	15
	25 days	5	4	A	Macerated Lesion Material	15
Experiment 3*	14 days	1	1	B	Controls—Segregated	16
	14 days	2	1	A	Controls—Within Pen	18
	14 days	3	2	A	Macerated Lesion Material	18
	14 days	4	3	A	Pure Cultures of *D*. *nodosus*, *Bacteroides spp*., *P*. *levii*, and *T*. *phagedenis*	18
	14 days	5	4	A	Pure Cultures of *T*. *phagedenis*	18
Experiment 4*	38 days	1	1	A	Macerated Lesion Material	48
	38 days	2	2	B	Pure Cultures of *D*. *nodosus*, *Bacteroides spp*., *P*. *levii*, *T*. *phagedenis*, *and C*. *urealyticus*	48
	38 days	3	3	A	Controls—Within Pen of Protocol 1	16
	38 days	4	3	B	Controls—Within Pen of Protocol 2	16
	38 days	5	3	C	Controls—Segregated	16
Experiment 5*	28 days	1	1	A	Macerated Lesion Material	48
	28 days	2	2	B	Macerated Lesion Material—frozen 24 hours	48
	28 days	3	3	C	Macerated Lesion Material—10:1 dilution	32
	28 days	4	4	D	Controls—Segregated	32
					**Totals:**	504

The pen designations denote whether animals were housed within the same pen or separate pens and the letters may designate different pens across experiments. The types of inoculum utilized for each of the different protocols is outlined in the manuscript text.

*experiments 2–5 all utilized foot abrasion for all protocols

Each of the four preliminary experiments was designed to examine specific questions regarding protocol optimization, while experiment 5 was designed to validate the finalized consensus protocol. Experiment 5 utilized forty Holstein steer calves which were housed as ten separate pens, each containing four calves. The location was Iowa State University Animal Research Station and the pens were located in 3-sided sheds with no access to areas without a roof. All four feet of each calf were enrolled and received the same inoculum. Negative controls for this study were isolated from all treatment groups with all four feet treated as controls. On day 0 of the trial, all feet were subjected to abrasion using a tungsten abrasion disk. A 5/8” diameter area of skin in the interdigital fold was abraded in a manner to remove the epidermis and approximately 50% of the thickness of the dermis. Following abrasion, a 4x4 gauze pad was soaked in Induction Broth which was a mixture of sterile growth media that contained 40% MTGE (Anaerobe Systems, Morgan Hill, CA), 30% Brain Heart Infusion Broth (BD and Company, Sparks, MD) 15% Trypticase Arginine Serine Broth [58], and 15% Mueller Hinton Broth (BD and Company, Sparks, MD). This gauze pad was placed over the abraded skin in the interdigital fold and wrapped with 2” Gorilla Tape, (Gorilla Glue Inc.), to minimize the transfer of moisture and debris into and out of the wrap. Calves were housed in their assigned pens and groups following the application of wraps and feet were monitored for side effects of the abrasion and wraps for 3 days.

On day 3 of the trial, inocula were prepared and administered to each of the feet. The inocula were prepared using tissue lesion biopsies from 14 adult cows with naturally occurring stage A1, A2, B1, B2, 3, and 4 digital dermatitis lesions (as described in the Iowa Digital Dermatitis scoring system [28]). A total of 20 grams of lesion material was harvested and placed into Induction Broth with the addition of 20% Fetal Bovine Serum (Sigma-Aldrich, St. Louis, MO). The lesions were combined and macerated in an anaerobic chamber using two scalpel blades, and 1.5 ml of the supernatant was placed into 3 ml syringes for inoculum #1. A second set of syringes was filled with 1.5 ml of the same inoculum and frozen at -80°C for 24 hours to serve as inoculum #2. A subset of the original inoculum was also diluted to 10% of the original concentration with the use of additional Induction Broth. A third set of syringes was filled 1.5 ml of diluted inoculum and served as inoculum #3. A final set of syringes filled with 1.5 ml of Induction Broth to serve as a control for inoculum #4. A 1” sterile plastic teat cannula (Jorgenson Labs, Loveland, CO) was placed on all of the syringes and they were packaged into a sterile Whirl-Pak bag (one per calf) under anaerobic conditions. Inocula were then deposited behind the wraps in the exact location that all of the feet were abraded using the sterile plastic teat cannula.

On days 11, 18, and 25 all wrapped feet were re-moistened by dispensing 1.5 ml of the Induction Broth behind each wrap in the location of abrasion using the same technique. On day 28 all wraps were removed and feet were photographed. At this time, all feet were biopsied using a 3 mm biopsy punch and treated with topical tetracycline.

Biopsies for histologic examination from all experiments were immediately placed in 10% neutral buffered formalin and stained with hematoxylin and eosin (H&E) using a Gemini AS Automated Slide Stainer (Thermo Fisher Scientific, Waltham, MA) with the H&E based staining protocol. Warthin-Starry staining was conducted using the Iowa State Veterinary Diagnostic laboratory standard operating procedure. A blinded pathologist evaluated the biopsies and categorized them into one of three pathologic grades as previously described [28]. Any lesions having a unique pathologic description were grouped in the “other” category. The grade 1 category encompassed all biopsies identified as normal bovine skin or granulation tissue. Grade 2 lesions were described as hyperkeratosis, acanthosis with surface hemorrhage and erythrocytic crusts. Grade 3 lesions were described as segmental localized necrotizing to necrosuppurative epidermitis with individual cell necrosis, ballooning degeneration of epithelial cells, necrotizing vasculitis, intralesional bacteria consisting of delicate spirochetes, bacilli, and coccobacilli.

Since all induced lesions were considered stage 3 based on the Iowa DD lesion scoring system, a new lesion scoring system was necessary to better define the severity of the lesions and allow for group comparisons. The lesions from all 21 experiments were macroscopically scored using a novel induced lesion scoring system developed exclusively for these experiments. The scoring system (Table 2) is a sum of these three criteria (size, color and evidence of healing) with more weight being given to the size of the lesion than the other two observations since this measure allows for easily demonstrating a progressive lesion development. The color of the lesion was used as a proxy for evidence of healing and re-epithelialization while the lesion edges score was used as a proxy for evidence of 2^nd^ intention healing. All photographs were blindly scored by a single observer (AK) with multiple photographs of each lesion examined in a random order. The correlation between the lesion scores and histologic changes consistent with digital dermatitis was highest when lesion scores were greater than or equal to 7 on the 10 point scale. For each individual foot to be designated as a successful induction, the macroscopic lesion score was required to be greater than or equal to 7.

**Table 2:** Macroscopic Lesion Scoring System.

DD Induction Lesion Scoring System:	
choose most accurate description from each of three observations and summarize (10 = most severe, 0 = no lesion)	
**Observation 1: Size of Lesion:**	
5	Expanding from initial abrasion area
3	No change in size
1	Lesion smaller than initial abrasion area
0	No lesion present
**Observation 2: Color of Lesion:**	
3	Bright red
2	Pink
1	White
0	No lesion present
**Observation 3: Edges of Lesion:**	
2	Non-descript edges
1	Well defined edges
0	No lesion present

The macroscopic induced lesion scoring system utilized by blinded observer to score the degree of lesion development. The total lesion score was the sum of all three observations. The macroscopic scoring system had a high level of correlation with the histopathologic changes associated with digital dermatitis, r (30) = .48, p < .01.

For all experiments, the unit of measure was the individual foot of each calf as for many of the experiments, each foot underwent a different protocol. All statistical analyses were done comparing groups within a given experiment as each experiment had a unique set of experimental conditions. To examine if difference existed between protocols within an experiment, a One-way ANOVA was conducted. To evaluate the differences between each of the groups, post-hoc t-tests were conducted assuming equal variances. As these comparisons involved multiple protocols per experiment, a Bonferoni correction was applied to each comparison. For the experiments in which microscopic and macroscopic scoring was done, a regression analysis was performed to evaluate the correlation between scores.

## Results

**Fig 1:**
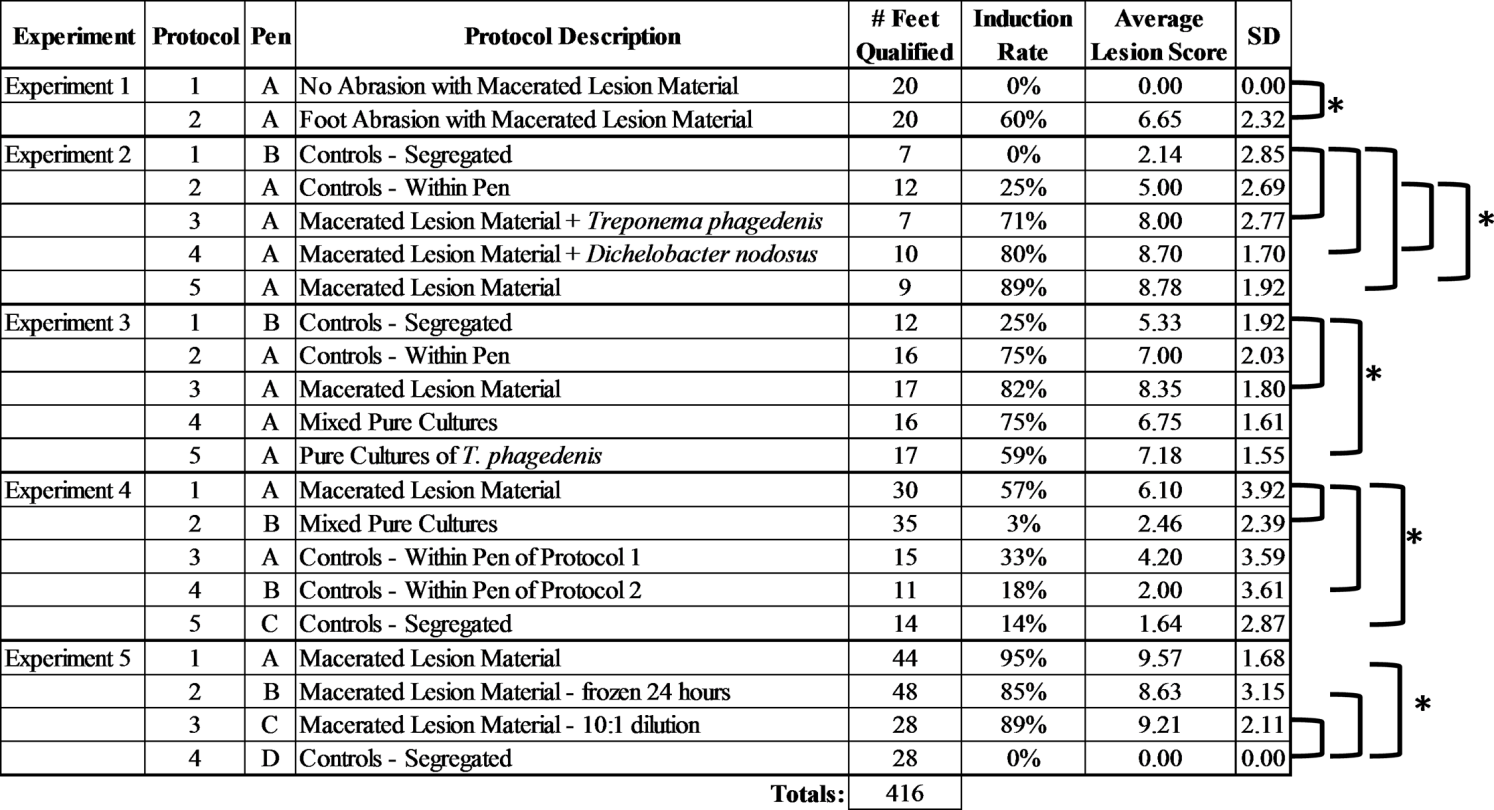
A summary of each protocol within the five experiments. The pen designations denote whether animals were housed within the same pen or separate pens and the letters may designate different pens across experiments. The number of feet qualified refers to the number of feet enrolled in each protocol that retained a wrap throughout the length of the study and did not receive topical or systemic antibiotics during the trial period. The induction rate is the percentage of feet that had a macroscopic lesion score of 7 or greater. The brackets denote statistical significance between groups with alpha = .05 and a correction for multiple testing.

A summary of the results from all 5 experiments is shown in Fig 1 (Data in S1 Table). Any calf that received antibiotics during the induction period of the trial or lost a wrap prior to the designated wrap removal date were not included in all statistical analyses or considered in the summary analysis.

### Experiment 1

Experiment 1 was designed to evaluate the necessity of skin abrasion in the induction protocol. Ten calves representing 40 feet were randomized to treatment group (abraded or non-abraded). At the time of wrap removal (7 days post abrasion) in our initial pilot study all wraps remained intact and all 40 feet qualified for analysis. Significant differences (p < .001) were noted between the protocol in which feet were abraded prior to induction and the protocol in which feet were inoculated without abrasion. The abrasion protocol had an average lesion score of 6.65, whereas the non-abraded feet had an average lesion score of 0.0. Based on our macroscopic scoring system cutoff of lesion score greater than or equal to seven for determination of successful induction, 12 of the 20 abraded feet (60%) were designated as having successful induction. In contrast, none of the non-abraded feet had induced lesions. Based on the results of this experiment we concluded that abrasion significantly improved induction success and included abrasion as part of all further induction efforts.

### Experiment 2

This experiment was designed to test two key factors in model development. First, we wanted to determine if addition of culture grown *Treponema phagedenis* or *Dichelobacter nodosus* to the macerated inoculum improved induction success. Second we wanted to determine if within-calf control feet could be used as a negative control group for lesion induction. In order to test the this issue we randomly assigned one foot per calf to receive skin abrasion, wrap and inoculation with sterile media while the other feet were included in treatment groups. A second segregated control group (i.e. all feet in pen were skin abraded, wrapped and inoculated with sterile media) was housed in a separate pen. For this experiment 18 calves representing 72 feet were enrolled. At the time of wrap removal, 45 of the original 72 wraps remained intact and qualified for analysis. When comparing the use of macerated lesion material (protocol 5), as done in experiment 1, the average lesion score (8.78) was statistically different (p < .01) to the within calf controls (5.00) and the segregated controls (2.14). The addition of pure cultures of *D*. *nodosus* and *T*. *phagedenis* in protocols 3 and 4 did not increase or decrease (p>.5) the average lesion score when compared to the use of macerated lesion material only in protocol 5. The average lesion scores of both protocols 3 and 4 were statistically different than the segregated controls (p < .01), although when compared to within calf controls, protocol 3 lesion scores (8.00) failed to reach statistical significance when compared to the lesion scores of this control group (5.00). The two control groups had noticeable differences between lesion scores with the within calf groups having an average lesion score of 5.00 and 25% of the feet having successful induction. In contrast the segregated controls had an average lesion score of 2.14 with 0% of the feet designated as successful induction. Despite these differences, the average lesion scores between the control groups did not reach statistical significance (p = .044) following a multiple comparison adjustment. Based on these results we made two important conclusions that influenced the development of the model. First, there was no evidence that the addition of culture grown *Treponema phagedenis* or *Dichelobacter nodosus* to the macerated inoculum improved induction success. Second we demonstrated that within-calf negative control feet (i.e. abraded, wrapped and sham inoculated with sterile media) had a 25% induction rate despite not being inoculated with bacteria and being continuously wrapped throughout the experiment. Two alternate hypotheses were developed based on this finding. Either the feet were becoming infected from within-pen environmental exposure penetrating the wrap, or the immune response of individual calves that developed in response to the challenged feet was resulting in lesion development in the control feet.

### Experiment 3

Experiment 3 was designed as a short duration pilot study (14 day wrap) to test the hypothesis that pure culture *Treponema phagedenis* alone or cocktails of pure growth DD associated bacterial consortiums could induce DD lesion development similar to that of macerated lesion material. Similar to experiment 2, both within-calf and segregated negative control groups were utilized. For this experiment 22 calves representing 88 feet were enrolled. At the time of wrap removal, 78 of the original 88 feet qualified for analysis as six wraps were lost and one calf (4 feet) was removed due to antibiotic treatment for respiratory disease. Similar to experiment 2, the macerated lesion protocol (protocol 3) had the highest induction rate (82%), and the highest average lesion scores (8.35). The lesion scores in the macerated group were statistically higher than those of the segregated control pen (5.33). However, the within pen control lesion scores (7.00) were not significantly different from any of the other protocols tested with the exception of the segregated control group. Protocol 4, which utilized the cocktail of culture grown organisms isolated from DD lesions, had an induction rate of 75% however the lesion scores were not statistically different from either of the control groups. The lesion scores from the pure culture of *T*. *phagedenis* (protocol 5) were statistically higher than those of the segregated control even though the induction rate was lower (59%) than that of protocol 4 (75%). Despite the fact that protocol 3 had a higher rate of induction and higher lesion scores compared to the other two treatment protocols that utilized culture grown organisms (4 and 5), protocol 3 lesion scores were not found to be significantly different than protocol 4 (p = .011) or protocol 5 (p = .049) with adjustments for multiple comparisons. Similar to experiment 3, we found that the within pen control group had significant lesion induction (75%) despite not being directly induced. From this experiment we concluded that inoculation of feet with pure growth organisms resulted in good induction of DD lesions when housed in the same pen as macerated lesion groups. However, the continued high level of induction in the within pen negative control groups suggested that there was still significant potential for alternative mechanisms of lesion induction in these groups.

### Experiment 4

Given the concerns over the potential for cross-contamination between the macerated induction and pure growth cocktail groups due to co-housing the animals, experiment 4 was designed to allow for segregation of the macerated lesion (protocol 1) group from the pure culture cocktail group (protocol 2). Similarly to the prior studies, a protocol utilizing macerated lesion material was tested as a means of comparing the induction success to the other experiments. In this experiment all 4 feet of each calf were inoculated with the same protocol and we dropped the within-calf negative control group, replacing it with a within-pen negative control group in which all four feet were sham inoculated. Finally, we had a segregated negative control group as in the two previous experiments. This design allowed us to segregate the calves into different pens based on the protocol. For this experiment 36 calves representing 144 feet were enrolled. At the time of wrap removal, 105 of the original 144 feet qualified for analysis as 35 wraps were lost and one calf (4 feet) was removed due to antibiotic treatment for respiratory disease. A large and significant difference was observed between the two treatment groups (p < .0001) with the macerated lesion protocol (protocol 1) lesion scores averaging 6.10 and the pure culture protocol (protocol 2) averaging 2.46. An even larger difference was observed on the number of successful inductions where protocol 1 was at 57% and protocol 2 was only 3%. Interestingly, protocol 1 was not statistically different (p = 0.12) from the control group that was housed within the same pen (average lesion score of 4.20), however, protocol 1 did reach statistical significance (p < .01) when compared to segregated control and the controls housed in the pen with the protocol 2 induction calves. Collective analysis for the results of this experiment yielded several important findings. First, the finding of a very high rate of lesion induction in the within-pen negative control group suggests that significant within pen cross-contamination of lesions was the source of lesion induction. The alternative hypothesis discussed in experiment 2 (i.e. that immune response was leading to the within-calf negative control lesion induction) could be excluded in this experiment due to the sham inoculation of all 4 feet in this group. Based on this finding and the poor lesion induction observed in the segregated cocktail inoculum group (protocol 2) of this experiment, we concluded that the success in previous experiments using pure growth cocktail for induction was likely due to cross-contamination from being housed in a contaminated environment.

**Fig 2:**
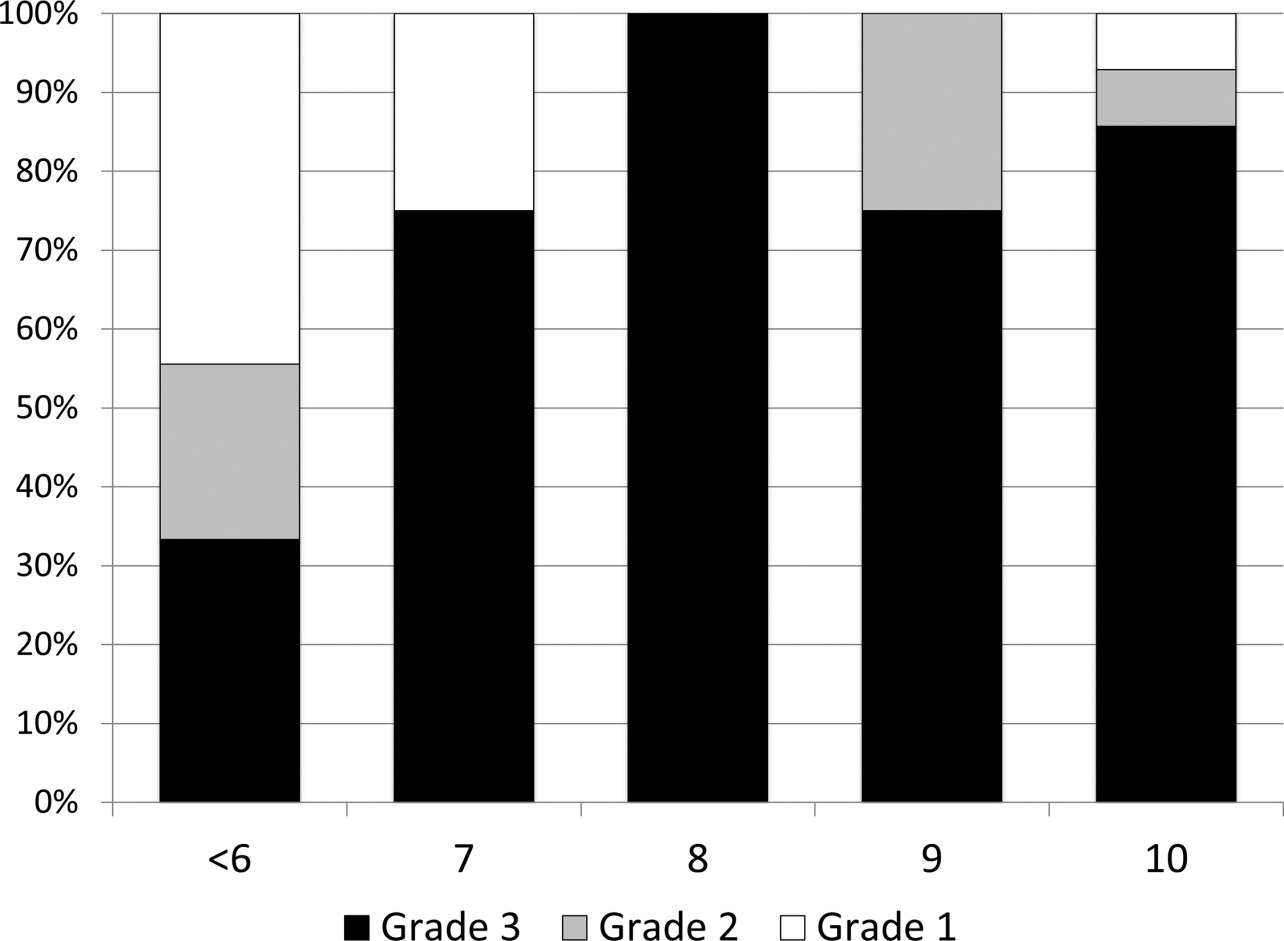
Lesion Grades for each Macroscopic Lesion Score. Histopathology score summary from each of the macroscopic scores. As biopsies were only taken from feet with visible lesions, the majority of the lesions scores were 6 or greater. Several lesion scores of 4 and 5 were included in the <6 category. The grade 1 category encompassed all biopsies identified as normal bovine skin. Grade 2 lesions were described as hyperkeratosis, acanthosis with surface hemorrhage and erythrocytic crusts. Grade 3 lesions were described as segmental localized necrotizing to necrosuppurative epidermitis with individual cell necrosis, ballooning degeneration of epithelial cells, necrotizing vasculitis, intralesional bacteria consisting of delicate spirochetes, bacilli, and coccobacilli.

The histological scoring of biopsies obtained at the time of wrap removal was analyzed for correlation to our macroscopic scoring system. There was a significant positive correlation between the macroscopic lesion score and the histologic grade designated, r(30) = .48, p < .01. A summary of histologic grades associated with each of the lesion scores is shown in Fig 2 (Data in S2 Table). As biopsies were only obtained from feet with any type of macroscopic lesion, the majority of the biopsies were from lesion scores 7–10. All lesion scores less than seven were lumped together for ease of presentation. For the lesion scores 7–10 the majority (19/23) were classified as histologic score Grade 3, whereas only three of the nine with scores less than seven were classified as Grade 3.

### Experiment 5

**Fig 3:**
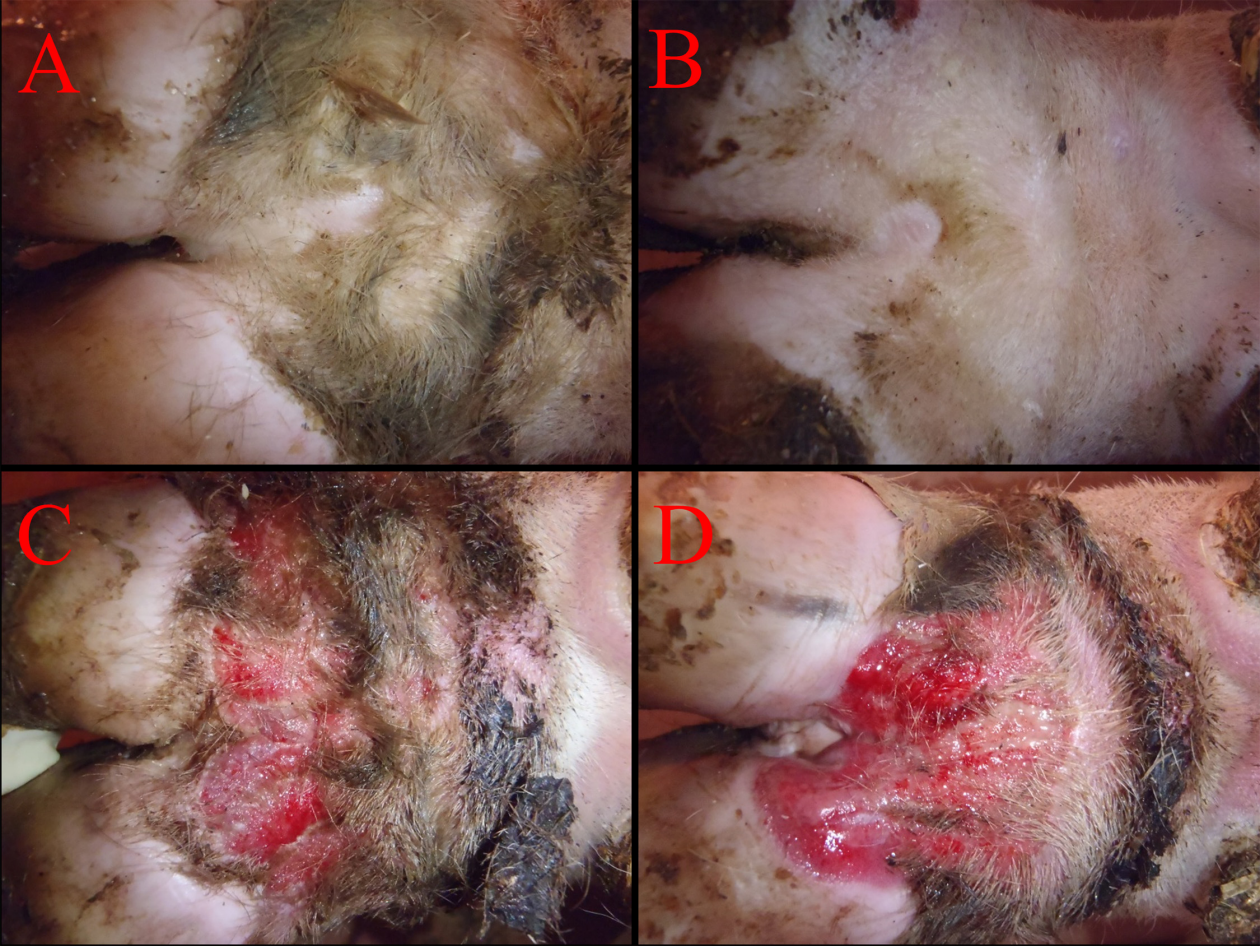
Representative DD Lesions from Experiment 5. Digital Dermatitis lesions on day 28 following wrap removal. Photos A and B represent control calves and photos C and D represent lesions that scored 10 using the induced lesion scoring system.

The final experiment was designed to validate the consensus induction protocol developed based on the outcomes of the first four experiments and to rule out a pen effect on induction. Since preparation of large volumes of macerated inoculum is time and labor intensive, we wanted to evaluate the induction success using frozen or diluted inoculum. As calves from each induction protocol were housed in separate pens, an accurate assessment of the true induction success as compared to the controls without the possibility of within-pen cross-contamination was possible. For this experiment 40 calves representing 160 feet were enrolled and at the time of wrap removal, 148 of the original 160 feet qualified for analysis with no wraps lost and three calves (12 feet) was removed due to antibiotic treatment for respiratory disease. At the conclusion of the trial, all three tested protocols (macerated-1, frozen-2, and dilute-3) had decidedly higher (p < .0001) lesion scores than the segregated negative controls. All three protocols had greater than 85% induction with average lesion scores higher than 8.5. Of the 28 control feet, there was not a single foot that had a lesion score greater than 0. Although lesion scores and percent induction were slightly higher in the typical macerated lesion protocol (1), there was no statistical difference between the other variations of macerated lesion material in protocols 2 and 3 indicating that a 90% dilution of inoculum or freezing of inoculum did not significantly decrease the effectiveness of the induction. Several examples of induced lesions that were scored as 10 using the induced lesion scoring system are shown in Fig 3.

**Fig 4:**
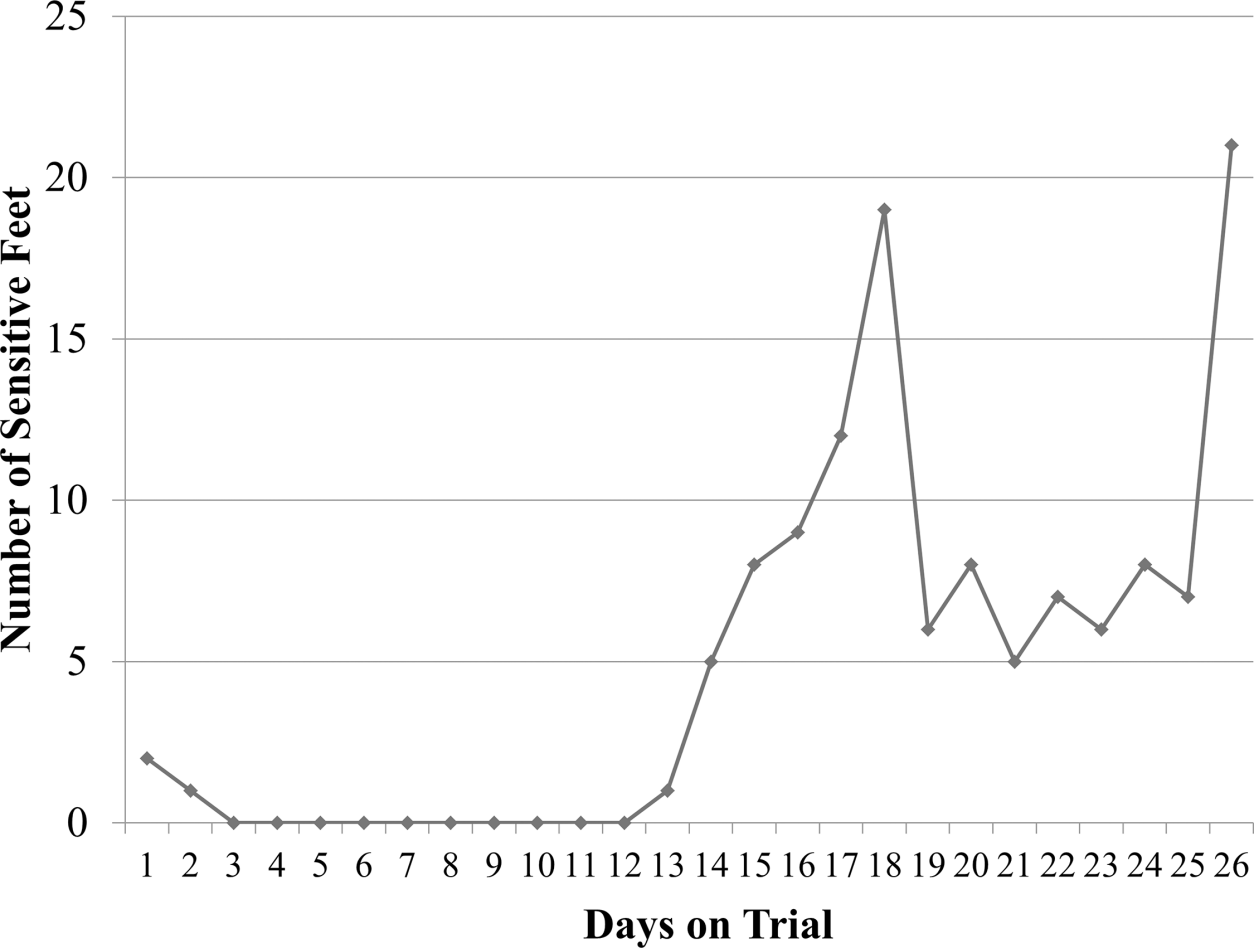
The number of sensitive feet for each day on trial for experiment 5. Feet were wrapped and abraded on day 0, inoculated on day 3 and wraps were taken off on day 28. The minimum objective measure for a sensitive foot was that a calf held the foot off the ground while standing.

Fig 4 shows the number of sensitive feet for each day of the trial (Data in S3 Table). The number of sensitive feet remained very low for the first 12 days of the trial with only three sensitive feet the first 2 days after abrasion. Approximately two weeks post abrasion the number of sensitive feet began to increase rapidly with an initial peak at day 19. This was followed by a period of time with lower numbers of sensitive feet until the end of the trial where the number of sensitive feet peaked again at day 26. There was a statistical difference in DD associated lameness between the different protocols (p < .05), with control feet having a significantly lower number of lame feet compared to all induction protocols (Table 3). There was also no statistical difference in lameness between the three induction protocols. A high level of correlation was also observed between macroscopic score and foot sensitivity (r(147) = .23, p < .0001) with 21% of feet with DD lesions showing signs of sensitivity.

**Table 3:** The Number of Lame Feet for Each of Protocol.

*Groups*	*n*	*Lame*	*Average*	*Std Err*
Control	28	0	0.0%	0.000
Frozen	48	6	12.5%*	0.048
Low Dose	27	7	25.9%*	0.086
Treatment	44	11	25.0%*	0.066

Lameness was defined as a foot that had a minimum of two observations of sensitivity in which at least one of them was a score of 2 or more.

*Indicates statistical significance when compared to controls

**Fig 5:**
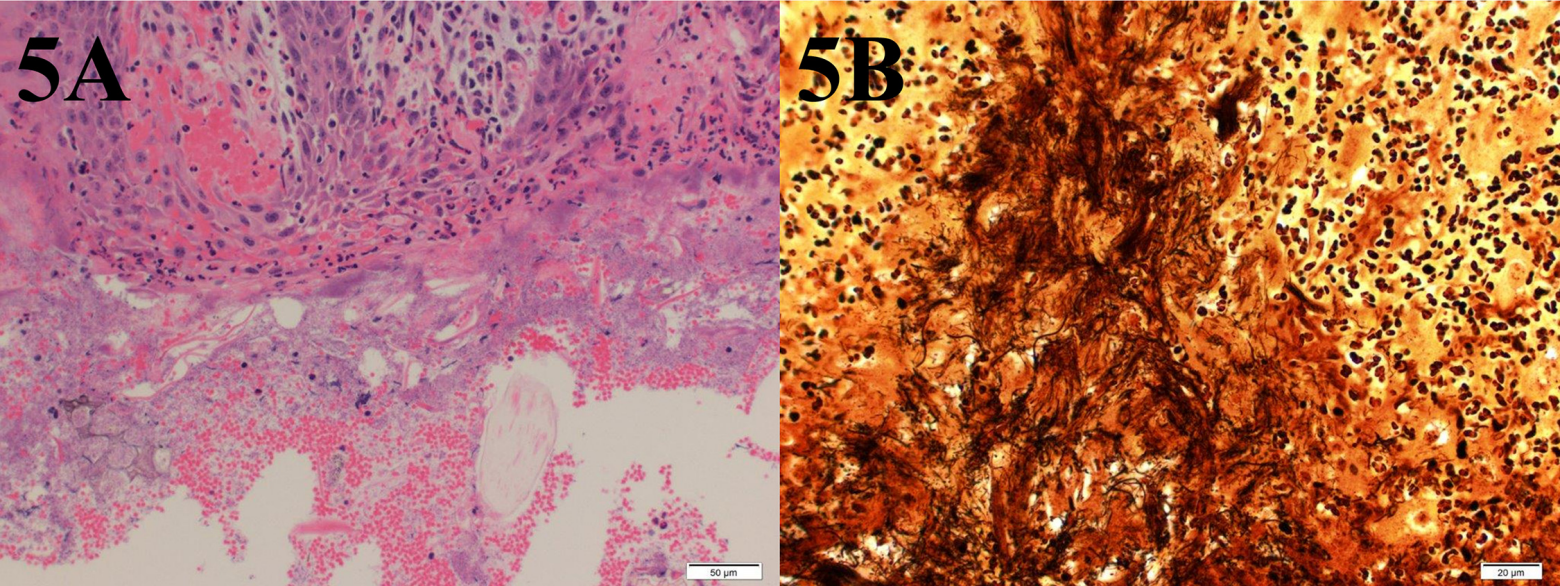
Grade 3 Digital Dermatitis Histopathologic Changes. 5A shows the characteristic changes of a Grade 3 lesion with necrosis of surface epithelium inflammatory infiltration, ballooning degeneration of epithelial cells, and colonizing bacteria within surface lesions. 5B shows more defined intra-lesional spirochetes within inflammatory loci when stained using W/S silver staining.

The histological scoring of biopsies obtained at the time of wrap removal was analyzed for correlation to our macroscopic scoring system (Data in S2 Table). Unlike experiment 4, all feet were biopsied regardless of macroscopic appearance of the skin. There was a much larger separation of lesion scores with the majority (140/147) of the scores either being 0 indicating normal skin or a lesion score of 9 or 10 indicating a large lesion. The lesion scores were highly correlated (r(145) = .87, p < .0001) to the histopathology scores with 98% of lesion scores 9 or 10 scored as grade 3 and 92% of lesion score 0 scored as Grade 1. An example of the histopathologic changes observed in Grade 3 lesions is shown in Fig 5.

## Discussion

The experiments presented in this manuscript represent the largest and most successful induction of digital dermatitis lesions ever reported. Using 504 feet enrolled in 21 unique protocols and a systematic approach we were able to develop a clinically relevant induction model. The model consistently produces DD lesions in naïve calves in less than one month, does not result in significant induction of disease in negative control calves, and can logistically be accomplished on a large enough scale to produce statistical significance. Furthermore, the lesions produced are located in an anatomical location consistent with naturally occurring DD lesions, and are macroscopically and microscopically indistinguishable from naturally occurring DD lesions. These collective attributes yield a model that can readily be applied to testing hypotheses regarding the pathophysiology of DD, evaluating vaccine efficacy and evaluating other treatment interventions.

The first four experiments were important in optimizing the final protocols presented in experiment 5. From experiment 1 we concluded that skin abrasion aided in rapid development of clinical DD lesions. However, the macroscopic appearance of the feet at seven days post abrasion made it difficult to determine the difference between naturally healing abraded skin and an effective induction, suggesting the need to prolong the period of wrap in future experiments and the need for an abraded negative control group. In experiment 2, an increased wrap duration was utilized and two sets of controls were included to evaluate the potential role of environmental exposure to digital dermatitis organisms infecting our within-pen negative control feet. Unfortunately, a number of wraps started falling off or needed to be removed between days 9 and 25 due to complications associated with the extremely cold weather causing our water resistant tape to become very non-pliable. Although not a statistical different, the two negative control groups demonstrated a large difference in percent induction and average lesion score. A similar experimental design was repeated in experiment 3 with a shortened wrap length of 14 days in an attempt to minimize the wrap associated difficulties. The shortened wrap length in this experiment did not allow sufficient time for our skin abrasions to heal sufficiently to effectively differentiate a healing abrasion from a digital dermatitis lesion. Despite the controls having higher lesion scores in experiment 3, the macerated lesion group and the pure cultures of *T*. *phagedenis* did reach statistical significance when compared to the segregated controls. However, similarly to experiment 2, the within calf controls were not statistically different from any of the other 3 protocols indicating some level of exposure to DD bacteria within the pen.

In experiment 4, we elected to make a fundamental change in the randomization protocol and study design. Based on the risk of cross-contamination observed in the previous experiment’s within-calf negative control group we opted to modify the design with calf level randomization (i.e. all four feet of a given calf received the same inoculum). This change also allowed for complete segregation of calves by treatment group. In order to further evaluate the role of cross-contamination we utilized a group of within-pen negative controls (all four feet sham inoculated) in each of the pens in addition to a segregated negative control group. As experiment 3 determined that a 14 day wrap was too short for consistently differentiating residual skin abrasion from digital dermatitis, it was elected to extend the wrap length in this experiment to 35 days. Although there were issues with maintaining wraps for prolonged periods of time in experiment 2, it was believed this was mostly due to the extreme cold during that trial. We also elected to drop down to a single injection of inoculum and follow up with additional sterile media to maintain wrap moisture throughout the prolonged length of the trial. In a modest improvement from experiment 2, 76% of the wraps remained intact until the end of the trial. Similarly to experiment 2, 100% of the wraps were intact on day 10, followed by a loss of approximately 5% of wraps per week until the end of the trial. The overall lesion scores across all groups were lower compared to the previous trials, but the macroscopic differentiation between skin abrasion and digital dermatitis lesions was much more defined. Unlike in experiment 3 when a statistical difference could not be realized between macerated lesion material protocol (1) and the pure growth organisms protocol (2), a very large difference (57% vs 3%, p < .0001) was found between these two protocols in this experiment when the groups were housed in separate pens. The macerated lesion protocol (1) again had the highest induction rate (57%) and lesion scores (6.10) compared to all other protocols and was considered statistically different than all protocols with the notable exception of the control calves housed within the same pen. This finding, coupled with the results from experiments 2 and 3, demonstrated that any feet housed within the same pen as a protocol that included macerated lesion material consistently had an increase in lesion scores and induction rate. This information calls into question previous reports claiming to have created a DD lesion with *Treponema* spp. culture broth, since those calves were commingled with other animals inoculated with DD homogenate [35].

Looking at the results of the first four experiments, it is apparent that the only protocol that consistently produced lesion scores that were statistically higher than controls were protocols utilizing macerated lesion material. The use of pure cultures was abandoned in the final experiment in favor of testing variations of the macerated inoculum protocol in an effort to identify means of simplifying the logistics of the study while also allowing for direct comparison between studies. As our trials increased in calf number, that amount of fresh lesion material became difficult to obtain within a short period of time necessary to make fresh macerate. In order to increase efficiency we hypothesized that use of a lower inoculum (10% of previous dose) or use of frozen macerate would provide acceptable induction rates. Furthermore, the ability to freeze inoculum would allow the use of a large single inoculum preparation in multiple studies, which would increase the consistency between experiments and allow for direct comparisons between experiments. The final wrap length for this trial was set at 28 days. This was based on the results the previous experiments indicating that 14 days was too short and 35 days was too long. Finally, in order to allow for assessment of pen level effects we elected to house animals in smaller pens (4 calves per pen) with multiple pens allocated per treatment group.

The results of experiment 5 provided the best induction results of the five experiments due to the systematic evaluation and adoption of protocol improvements during the first 4 trials. The lesion scores of all three treatment protocols in experiment 5 were significantly higher (p < .0001) than the negative control calves. Protocol 1 (standard dose, fresh macerated DD lesion) had the highest lesion scores (9.57) and percent induction (95%) of any of the 21 protocols reported here. There was a slight decrease in lesion scores with the frozen and 10% inoculum groups, although it was not found to be statistically significant than protocol 1. This indicates that the amount of inoculum needed to induce DD lesions was far less than originally anticipated. This may have been a contributing factor in the prior experiments where feet in the same pen as macerated lesion protocols were developing DD lesions from apparent environmental exposure. The wrap duration of 28 days utilized in this experiment provides the ideal balance between allowing the skin abrasions to fully heal (making it easier to identify true DD lesions) and minimizing the complications associated with the long-term wraps. As a result, all 28 negative control feet had lesion scores of 0, further supporting our observations of possible environmental exposure resulting in cross contamination between protocols when commingled. The wrap integrity in this trial was far better than in prior long term protocols, with all wraps lasting the full 28 days of the experiment. Several factors were believed to play a role in the success of these wraps. From prior experiments, slight differences were observed in the way wraps were applied. Slight adjustments to how the feet were wrapped including wrapping further down on the claw and loosening the tops of the wraps made a large difference in not irritating the skin requiring wrap removal. Another factor that likely contributed to the difference is the change in housing. The calves were kept in 10’ x 10’ pens that were completely covered. Whereas in prior experiments the calves were in pens 100’ x 20’ that had a covered area, but also had a dirt area. The mud and manure in the larger pens would cause many of the wraps to be pulled off when the calves would run in the pens. The smaller pens decreased the activity of the calves and the clean and controlled environment allowed for wraps to remain intact for the entire length of the trial.

The inclusion of lameness as an objective measure for induction of DD lesions was important to provide a clinical measure of lesion induction. The use of locomotion score in natural DD lesions has been shown to be unreliable with only 26% of cows with slight lesions and 39% of cows with severe DD lesions [59] shown to have locomotion scores of 3 or greater on a five point scale [57]. The use of locomotion score also eliminates the ability to assess each limb individually. Therefore, we felt that it was necessary to develop an objective measure for each foot subjected to induction. The results of this induction show a similar level of lameness compared to naturally developing lesions with 21% of the feet showing signs of sensitivity. This is similar to the 26% of cows with DD lesions showing signs of lameness found by Frankena et al [59]. The spike of sensitive feet at day 19 of the study initiated our IACUC protocol which required calves with a locomotion score of 4 out of 5 to be treated with meloxicam. The use of an NSAID in these cases provided analgesia, but also decreased the amount of swelling in the foot. We believe that this decrease in swelling under the wrap decreased the sensitivity of the lesions due to less pressure being applied directly to the lesion surface. As seen in Fig 4, the number of sensitive feet decreased rapidly following administration of pain medication and remained constant until near the end of the study. The reason for the spike in foot sensitivity near the end of the study is likely 2-fold, 1) the DD lesions induced were becoming more severe and associated with increased lameness and 2) the growth of the feet during the trial was beginning to create increased pressure under the wrap. The results of our foot sensitivity measurements correlate well with macroscopic and histopathologic lesion scores and provide additional assurances to an induction of DD lesions similar to naturally developing lesions.

Valuable information and insights into DD induction were obtained from the first four experiments and integration of that knowledge into a final consensus protocol led to the highly successful induction described in experiment 5. We believe that several important premises have emerged from this work that are pertinent to induction of DD lesions. First, use of dairy calves provided consistent induction success and allows for known DD history on each animal in the study. This conforms to the guidelines of the USDA APHIS Biologics Regulations and Guidance for an ideal induction model utilizing immunologically naïve animals. Furthermore, dairy calves are more economical, easier to house and easier to restrain during induction trials, all of which allows for easier scale-up of induction trials. Most of our trials utilized 35–40 calves, however the ability to use a lower dose of frozen macerate allows for even further expansion of those numbers if necessary. Second, experiment 1 clearly demonstrated that abrasion of skin prior wrapping feet was vitally important to the success of rapid lesion induction. Third, the duration that feet were wrapped was a very important factor in the success of each experiment with approximately 28 days being the ideal timeframe. Shorter wrap-length times did not allow the skin abrasions to fully heal, making macroscopic differentiation of these to true DD lesions difficult to assess. Longer durations than 28 days was associated with side effects due to constriction of the wrap associated with calf growth. Fourth, the highly infectious nature of the disease process was also demonstrated by the fact that commingling of calves within pens resulted in a high level of cross contamination. This is especially interesting given the fact that both the induced lesions and the negative control feet were wrapped for the entirety of the study. Therefore, this provides strong evidence that direct contact with lesions is not required for disease transmission, and that the infectious dose is small enough to leak out of one wrap and into another wrap with relatively high frequency. The low infectious dose is also supported by our success using a lower dose and frozen inoculum. Collectively these points confirm that it is important for all DD induction trials to have appropriate controls and segregation in place to assure that cross contamination does not confound interpretation of the results.

The DD induction model described has undergone significant refinement and provides substantial benefits compared to the previously described DD induction model. [35] First, the scale of experimental validation is considerably different with our experiments utilizing a total of 504 feet from 126 three-month-old calves, compared to eight feet from four yearling heifers. Second, our protocol can rapidly induce a large number of lesions as evidenced by the final experiment inducing lesions on 108 of 120 (90%) feet within 28 days as compared to a 63-day protocol. Third, the DD lesions produced in this study more accurately mirror naturally occurring DD lesions in terms of anatomic location and severity, whereas the previously described model was only able to induce lesions near the dew claws.

Perhaps the most significant differences between the two protocols relate to the preparation and management of the feet and wraps prior to induction. We replaced the preliminary 18-day water maceration step with a skin abrasion step in our study. While both protocols induce an artificial manipulation of the foot that predisposes to lesion development in a manner quicker than natural disease, our model does this in 3 days versus 18 days. Our protocol also does this in a single time-point upon application of the wrap, whereas the previous model requires refilling the rubber boot on each foot with water every 12 hours for 18 days prior to induction. While one could potentially develop a model that doesn’t manipulate the integrity of the skin, (as we did in experiment 1) lesion development is much slower with a lower induction rate at a significantly increased time and financial cost. The complexity of the wrap was also significantly different between the studies with our protocol involving a 4x4 and moisture resistant tape that was applied and remained on for the length of the study versus wraps that involved inoculation chambers, cotton, seven layers of plastic wrap, a rubber boot and duct tape that was applied and removed up to 12 times during the study. We felt that creating a microaerophilic environment behind the wrap and maintaining that environment without removing the wraps was vital to the protocol. These simple and effective techniques utilized in our experiments greatly reduced the cost per calf, increased the number of animals that can be induced in a single study, and decreased the duration of housing for the calves on study allowing for the type of large scale trials necessary for treatment and prevention trials.

In conclusion, the protocol outlined in this study has the potential to be useful in a number of future research investigations. Research with novel vaccines against DD would be the most obvious use of the induction model. Alternate uses of this model could also include the testing of candidate pathogens isolated from DD lesions for their ability to reproduce disease. The ability to rapidly produce a large number of DD lesions in young animals also makes the model ideal for testing new treatment strategies. The use of this model in treatment trials has several advantages. First, the use of young calves makes handling calves and feet much easier. Second, as these animals are not lactating and being moved daily to and from a parlor, it is easier to eliminate any environmental biases induced from a common exposure area. Third, there is a known level of chronicity within the induced lesions as well as a similar morphology that makes treatment outcomes easier to define and measure. Fourth, the use of induced lesions eliminates the time and cost associated with identifying natural lesions with a herd to use for a treatment trial. Finally, we feel that this model’s simplicity and consistency of induction gives it the ability to be scaled up to meet statistical significance for any number of the aforementioned research trials.

## Supporting Information

S1 File Supplemental materials and methods for experiments 1 through 4. This file contains detailed materials and methods for the first four experiments.
(DOCX)

S1 Table Calf lesion scores for all five trials. This file is an excel file of all individual calf lesions scores from each of the five experiments. Each experiment is a different sheet in the file.
(XLSX)

S2 Table Calf level histopathiology data for experiments 4 and 5. Excel file of calf level histopathology data. Each experiment is a different sheet in the file.
(XLSX)

S3 Table Calf daily lameness scores. Daily lameness recorded data.
(XLSX)
