# Supplementary material for: A Highly Effective Protocol for the Rapid and Consistent Induction of Digital Dermatitis in Holstein Calves
Source: PLoS One. 2016 Apr 27;11(4):e0154481. doi: 10.1371/journal.pone.0154481 (PMC4847800; doi:10.1371/journal.pone.0154481)
Supplement: S1 File — This file contains detailed materials and methods for the first four experiments. (DOCX) [file pone.0154481.s001.docx]

Supplemental File – Preliminary Induction Trials

Materials and Methods:

Experiment 1:

Ten Holstein steer calves were utilized for this study. The calves were housed in a single group at the Iowa State University Veterinary Medicine Research Institute in a 3-sided shed with an outdoor run. For this experiment, all four feet were utilized from each of the calves. For each calf, feet were randomly assigned as to have two feet per calf for each protocol either subjected to abrasion at the onset of the trial or to be wrapped without abrasion. As this was a comparison between two variables, no controls were utilized for this experiment. On day 0 of the trial, all feet were subjected to abrasion using a tungsten abrasion disk. A 5/8” diameter area of skin in the interdigital fold was abraded in a manner to remove the epidermis and approximately 50% of the thickness of the dermis. Following abrasion, a 4x4 gauze pad was soaked in Induction Broth which was a mixture of sterile growth media that contained 40% MTGE (Anaerobe Systems, Morgan Hill, CA), 30% Brain Heart Infusion (BHI) Broth (BD and Company, Sparks, MD) 15% Trypticase Arginine Serine (TAS) Broth ([1](#_ENREF_1)), and 15% Mueller Hinton Broth (BD and Company, Sparks, MD). This gauze pad was placed over the abraded skin in the interdigital fold and wrapped with 2” Gorilla Tape, (Gorilla Glue Inc.), to minimize the transfer of moisture and debris into and out of the wrap. Calves were housed in their assigned pens and groups following the application of wraps and feet were monitored for side effects of the abrasion and wraps for 3 days.

On day 3 of the trial, an inoculum was prepared and administered to each foot wrapped on day 0. The inoculum was prepared using biopsies from four adult cows with stage 3 and 4 DD lesions as described in the Iowa DD scoring system ([2](#_ENREF_2)). Approximately five grams of lesion material was harvested and placed into MTGE Broth. The lesions were combined and macerated in an anaerobic chamber using two scalpel blades, and 1.5 ml of the supernatant was placed into each of 40 three ml syringes. A 1” sterile plastic teat cannula (Jorgenson Labs, Loveland, CO) was placed on the syringes and they were packaged into a sterile Whirl-Pak bag per calf under anaerobic conditions. The inoculums were deposited behind the wraps in the exact location in which 50% of the feet were abraded. On day 7 the wraps were removed and feet were photographed. Lesion progression was monitored via twice weekly photographs and locomotion scores until the conclusion of the study on day 32

Experiment 2:

Eighteen Holstein steer calves were utilized for this study. The calves were housed in the same location as experiment 1. Three calves served as control calves and were placed in a completely separate pen from the rest of the calves. Similar to Experiment 1, all four feet were utilized from each of the calves. One foot from each of the induction calves served as a within calf control for this experiment. The other 3 feet from each calf were randomly assigned to one of three inoculums. All feet in this experiment were abraded and wrapped on day 0 in an identical manner as that described in Experiment 1. Calves were allowed to commingle following the application of wraps and feet were monitored for side effects of the abrasion and wraps.

On day 2 of the trial, inoculums were prepared and administered to each of the feet wrapped on day 0. The inoculums were prepared using biopsies from three adult cows with stage A2 and stage 4 DD lesions. Approximately five grams of lesion material was harvested and placed into MTGE Broth, macerated in an anaerobic chamber, and 1.5 ml of the remaining supernatant was placed into 15 three ml syringes for inoculum #4. A separate set of 15 syringes was filled with 0.75 ml of the macerated lesion inoculum and 0.75 ml of MTGE Broth containing log growth of 3 isolates of *Treponema phagedenis*. The isolates of *T. phagedenis* were isolated in our laboratory from cases of digital dermatitis in adult Holstein dairy cows. Previous work in our lab showed log growth at approximately 72 hours with an estimated 10^7^ live cells per ml of MTGE Broth. This 50% mixture of macerated lesion and *T. phagedenis* organisms was used as inoculum #2. A third set of 15 syringes was filled with 0.75 ml of the macerated lesion inoculum and 0.75 ml of MTGE Broth containing *Dichelobacter nodosus* (ATCC® 25549™). The D*. nodosus* was 48 hour growth on BRU Agar (Anaerobe Systems, Morgan Hill, CA). Three plates of lawn growth were washed with TAS broth and added to the macerated lesion inoculum in a 50% mixture to make inoculum #3. Previous work in our lab showed this technique yielding approximately 10^9^ live cells per ml of TAS broth. An additional set of 15 syringes were filled with 1.5 ml of MTGE Broth to serve as the within calf controls. A final set of 12 syringes were filled with 1.5 ml of MTGE Broth (inoculum #1) for all 4 feet of the three segregated control calves. A 1” plastic teat cannula was placed on all of the syringes and they were packaged into a sterile Whirl-Pak bag per calf under anaerobic conditions. The inoculums were again deposited behind the wraps in the exact location that all of the feet were abraded.

On day 4 of the trial, inoculums were prepared in an identical manner to what was done on day 2 with approximately five grams of macerated lesions was collected from four adult dairy cows with stage A2, B2, 3, and 4 DD. The inoculums were again deposited behind the wraps in the exact location in where all of the feet were abraded. Following induction, feet were re-moistened using MTGE Broth containing 33% Fetal Bovine Serum (Sigma-Aldrich, St. Louis, MO) on days 10 and 18. On day 24 all wraps were removed and feet were photographed. Lesion progression was monitored via twice weekly photographs and locomotion scores until the conclusion of the study on day 49. At the conclusion of the study, any remaining visible lesions were biopsied using a 3 mm biopsy punch and treated with topical tetracycline until no visible lesions remained.

Experiment 3:

Twenty two Holstein steer calves were utilized for this study. The calves were housed in the same location as experiment 1. Four calves served as control calves and were placed in a completely separate pen from the rest of the induction calves. Similar to Experiments 1 and 2, all four feet were utilized from each of the calves. One foot from each of the induction calves served as a within calf control for this experiment. The other 3 feet from each calf were randomly assigned to one of three inoculums. All feet in this experiment were abraded and wrapped on day 0 in an identical manner as that described in Experiment 1. Calves were allowed to commingle following the application of wraps and feet were monitored for side effects of the abrasion and wraps.

On day 3 of the trial, inoculums were prepared and administered to each of the feet wrapped on day 0. The inoculums were prepared using biopsies from three adult cows with stage A2 and 4 digital dermatitis lesions. Approximately five grams of lesion material was harvested and placed into MTGE broth, macerated in an anaerobic chamber, and 1.5 ml of the remaining supernatant was placed into 18 three ml syringes for inoculum #2. A separate set of 18 syringes (inoculum #4) was filled with 1.5 ml of MTGE broth containing 72 hour log growth of the same 3 isolates of *Treponema phagedenis* utilized in Experiment 2. A third set of 18 syringes was filled with 1.5 ml of mixed pure cultures isolated from DD lesions in adult Holstein dairy cattle. This mixture contained *Dichelobacter nodosus, Bacteroides spp., Porphyromonas levii,* and *Treponema phagedenis*. The *D. nodosus* was prepared in an identical manner as described in Experiment 2 and contributed to 15% of the inoculum. Four isolates of *Bacteroides* spp. and four isolates of *P. levii* were prepared from 48 hour growth on BRU Agar. These plates of lawn growth from each bacterium were washed with BHI Broth and each contributed to 15% of the inoculum. The final 55% of the inoculum was from the 72 hour growth of the three isolates of *T. phagedenis* described in inoculum #2. This mixture of organisms isolated from DD lesions made up inoculum #3. Previous work in our lab showed this technique yielding approximately 10^9^ live cells per ml of *D. nodosus, Bacteroides*, and *P. levii*. An additional set of 18 syringes were filled with 1.5 ml of Induction Broth which contained 30% BHI, 15% TAS, and 55% MTGE to serve as the within calf controls. A final set of 16 syringes were filled with 1.5 ml of Induction Broth for all 4 feet of the four segregated control calves. A 1” plastic teat cannula was placed on all of the syringes and they were packaged into a sterile Whirl-Pak bag per calf under anaerobic conditions. The inoculums were again deposited behind the wraps in the exact location that all of the feet were abraded.

On day 10 of the trial, inoculums were prepared in an identical manner to what was done on day 3. Approximately five grams of macerated lesions was collected from seven adult dairy cows with stage A1, A2, B1, B2, 3, and 4 digital dermatitis lesions as described in the Iowa Digital Dermatitis scoring system to be utilized in the inoculums. The inoculums were again deposited behind the wraps in the exact location in where all of the feet were abraded. On day 24 all wraps were removed and feet were photographed. Lesion progression was monitored via twice weekly photographs and locomotion scores until the conclusion of the study on day 38. At the conclusion of the study, any remaining visible lesions were biopsied using a 3 mm biopsy punch and treated with topical tetracycline until no visible lesions remained.

Experiment 4:

Thirty six Holstein steer calves were utilized for this study. The calves were housed in three separate groups. The main treatment groups were housed in two separate pens each containing 16 calves at the Iowa State University Veterinary Medicine Research Institute in a 3-sided shed with an outdoor run, whereas the four controls were moved to a completely naïve location within the Iowa State University Teaching Hospital. Similar to Experiments 1, 2, and 3, all four feet were utilized from each of the calves. For Experiment 4, all four feet of each calf would be treated with the same inoculum and there would not be a within calf control. A subset of 4 calves from each of the groups of 16 would serve as within pen controls. All feet in this experiment were abraded and wrapped on day 0 in an identical manner as that described in Experiment 1. Calves were allowed to commingle following the application of wraps and feet were monitored for side effects of the abrasion and wraps.

On day 3 of the trial, inoculums were prepared and administered to each of the feet wrapped on day 0. The inoculums were prepared using biopsies from eight adult cows with stage A1, A2, B1, B2, and 4 DD lesions. Approximately 20 grams of lesion material was harvested and placed into Induction Broth containing 40% MTGE, 30% BHI, 15% TAS, and 15% Mueller Hinton Broth, macerated in an anaerobic chamber, and 1.5 ml of the remaining supernatant was placed into 48 three ml syringes for inoculum #1. A second set of 48 syringes was filled with 1.5 ml of mixed pure cultures isolated from DD lesions in adult Holstein dairy cattle. This mixture contained *Dichelobacter nodosus, Bacteroides spp., Porphyromonas levii, Campylobacter urealyticus,* and *Treponema phagedenis*. The *D. nodosus, Bacteroides spp.,* and *P. levii,* were all prepared in an identical manner as described in Experiment 3 and each contributed to 15% of the inoculum. An additional 40% of the inoculum was from the 72 hour growth of the three isolates of *T. phagedenis* described in Experiment 3. The final 15% of *C. urealyticus* was prepared from two isolates each plated on sheep blood agar and incubated under microaerophilic conditions for 72 hours. The plates were washed with Mueller Hinton Broth and contributed to approximately 15% of the final Induction Broth. Previous work in our lab showed log growth at approximately 72 hours with an estimated 10^7^ live cells per ml of BHI Broth. A final set of 48 syringes were filled with 1.5 ml of Induction Broth to serve as controls. Sixteen of the syringes were used on all 4 feet of the four segregated control calves, 16 were used on all 4 feet of four control calves within the same pen as those receiving inoculum #1, and the final 16 were used on all 4 feet of four control calves within the same pen as those receiving inoculum #2. A 1” plastic teat cannula was placed on all of the syringes and they were packaged into a sterile Whirl-Pak bag per calf under anaerobic conditions. The inoculums were again deposited behind the wraps in the exact location that all of the feet were abraded.

On days 11, 17, and 25 all wrapped feet were re-moistened by dispensing 1.5 ml of the Induction Broth behind each wrap in the location of abrasion. On day 37 all wraps were removed and feet were photographed. At this time, any remaining visible lesions were biopsied using a 3 mm biopsy punch and treated with topical tetracycline until no visible lesions remained.

1. Skerman, T.M., *Determination of some in vitro growth requirements of Bacteroides nodosus.* J Gen Microbiol, 1975. **87**(1): p. 107-19.

2. Krull, A.C., et al., *Deep sequencing analysis reveals temporal microbiota changes associated with development of bovine digital dermatitis.* Infect Immun, 2014. **82**(8): p. 3359-73.
